# Spent Brewer’s Yeast as a Source of Insoluble β-Glucans

**DOI:** 10.3390/ijms22020825

**Published:** 2021-01-15

**Authors:** Ionut Avramia, Sonia Amariei

**Affiliations:** Faculty of Food Engineering, Stefan cel Mare University of Suceava, 720229 Suceava, Romania; sonia@usm.ro

**Keywords:** particulate β-glucans, spent brewer’s yeast, *Saccharomyces cerevisiae*, bioactive polysaccharides

## Abstract

In the brewing process, the consumption of resources and the amount of waste generated are high and due to a lot of organic compounds in waste-water, the capacity of natural regeneration of the environment is exceeded. Residual yeast, the second by-product of brewing is considered to have an important chemical composition. An approach with nutritional potential refers to the extraction of bioactive compounds from the yeast cell wall, such as β-glucans. Concerning the potential food applications with better textural characteristics, spent brewer’s yeast glucan has high emulsion stability and water-holding capacity fitting best as a fat replacer in different food matrices. Few studies demonstrate the importance and nutritional role of β-glucans from brewer’s yeast, and even less for spent brewer’s yeast, due to additional steps in the extraction process. This review focuses on describing the process of obtaining insoluble β-glucans (particulate) from spent brewer’s yeast and provides an insight into how a by-product from brewing can be converted to potential food applications.

## 1. Introduction

Worldwide in 2018 about 1.9 billion hL of beer was produced. Of this volume, the European Union produced more than 390 million hL of beer and is considered the second largest beer producer in the world after China [1,2]. It has the capacity to generate as by-products approximately 0.9 million tons of spent brewer’s yeast (up to 4 kg/hL of beer) [3], while other studies indicate values up to 2.3 kg/m^3^ of yeast [4]. The data vary depending on the dry matter content or citation collected.

Mainly used in animal feed, the application of various pretreatment processes can make residual yeasts a product with multiple uses for human consumption as follows: enhance or impart a meaty flavor to food [5,6]; an alternative protein source [7]; folate-rich yeast extract (depending on the type of yeast) [8]; as a water-insoluble polysaccharide, such as β-glucan with increased potential in the pharmaceutical and food industry [9,10].

β-glucans are polymers of glucose with different types of glycosidic linkages and anomeric configurations [11]. These glucans act as a barrier with the main role in supporting the cell wall due to a rigid structure of β-D-glycosidic bonds that are interconnected with other glucose chains, particularly through the β-1,3 and β-1,6 bonds [12]. They are also used for medical purposes due to a strong effect on the immune system [13]. Unlike cellulose, which is a polymer of β-1,4 glucose [14], fungal glucans differ substantially in structure by the anomeric α or β covalent position [15]. Discriminatory in the analysis of polysaccharides are the molecular mass and spatial conformation [16].

Thus, insoluble β-glucans found in yeast refers to those that have a fibrous backbone consisting of β-1,3-glucan chains branched to β-1,6-glucose polymers. Of these glucans, other components of the wall are bound with low content in chitin or mannoproteins [17]. By using appropriate isolation procedures (keeping the temperature low and pH within the limits) the triple helix conformation shown to induce cytokine stimulation can occur [18,19].

## 2. Yeast Cell

*Saccharomyces* are the most important genus for the food industry including species such as *Saccharomyces cerevisiae* with different strains used in bakery, wine or beer making [20]. *S. paradoxus*, the closest relative to *S*. *cerevisiae*, recently proven to have good brewing abilities [21] whereas *S. bayanus* is used in lager beer and *S. uvarum* for wine and cider [22]. This single cell organism is capable of rapid multiplication by budding and possesses a widely exploited genetic system [23]. Multiplication occurs asymmetrically, thus the production of glucans and mannoproteins appear at the level of bud scars and spread over the entire cell surface [24].

As a model to study eukaryotic cells, *Saccharomyces cerevisiae* is generally recognized to be safe, and has a simple genetic manipulation system consisting of a genome of 5800 genes encoding proteins in 12 pairs of DNA [25]. *Saccharomyces cerevisiae* produces spherical and ellipsoidal cells with a size range between 1–7 µm wide and up to 5–10 µm long. Intensively studied, *Saccharomyces cerevisiae* is protected by a thick and mechanically resistant cell wall mostly made from polysaccharides and glycoproteins [26,27] of which 50–55% is represented by β-1,3-glucans, and up to 15% is composed of β-1.6-glucans [27,28]. The thick wall delimits the plasma membrane inwards through the cytoplasmic matrix. Intracellular content includes, amongst others, a nucleus, storage organelles, the Golgi apparatus, endoplasmic reticulum, vacuoles, enzymes, lysosomes.

Regarding the complexity of the yeast cell, this can be simplified by referring to the three main constituents: cell wall, cytoplasm and nucleus. The cell wall has mechanical properties of E = 0.75 ± 0.06 MPa (through a local measurement of the Young’s modulus by nondestructive methods of atomic force microscopy) [29], which is significantly higher than that of bacteria (E = 98 kPa) [30]. This indicates that β-glucans from yeast are more difficult to extract. However, their health benefits are considerable.

### 2.1. Mechanical Strength of Yeast

The exact shape of this single-cell organism is difficult to generalize especially in brewing. This is due to serial fermentation that occurs 8–15 times [31,32]. Cell division occurs asymmetrically. Bud scars emerge five or more times on the cell surface (up to 50 times according to Bühligen et al.) [32] making them wrinkle starting from the third generation [33]. Therefore, stress and growth conditions have a direct impact on the integrity and strength of the cell wall. This compels us to use mathematical models to assess cell dynamics and functions such as surface stiffness, density or Young’s modulus, which helps deduce the mechanical properties of yeast. Generally accepted in the description of the mechanical properties, elastic modulus requires knowledge of the wall thickness (using transmission electron microscopy) which can be calculated as a ratio between thickness and cell radius during compression tests [34]. Thus, the mechanical properties after compression of a single cell until it bursts (between 80–104 µN applied force) changed dramatically from 170 MPa for a daughter cell to a mean of 460 MPa after three or more bud scars on the cell surface [34]. This is, perhaps, due to the chitin content that is formed mainly on the scar rings after cell separation, accounting for up to 2% of the whole cell [23,35]. The cell stiffness, by measuring 28 cells without suffering morphological changes through multiplication, after the application of 268 repeated cycles of force, decreased from an average of 5.7 N/m to 0.85 N/m This indicates a degradation of the cell wall components [36]. Zarandi et al., using finite element analysis of the yeast, deduced that spherical cells that can reach values up to 9 µm (with an elastic wall of 200 nm) have a cytoplasmic rigidity of 10^4^ N/m^2^ and a Poisson’s ratio of 0.499 (assuming zero-value displacement constraints on one node at the bottom of the cell) [37]. Other studies involving postdiauxic cells (grown in the presence of two carbon sources) indicated that the cell wall is more resistant to the action of glucanases compared to those cultivated on rich media, caused by a low permeability of the cell wall [38].

### 2.2. Cell Wall

A considerable amount of energy is invested through the biogenesis of the cell wall, which comprises up to 25% of the cell weight (depending on the growth conditions) [39], [40]. The synthesis is directed by the so-called cell wall integrity signaling (CWI) [41] that largely occurs in the formation of glucan content [42]. It is known that in the fermentation process of beer, yeasts use for biogenesis approximately 10% of fermentable sugars from the environment [43]. This energy is stored in a 200 nm thick wall in the form of carbohydrates, which are of high interest in food processing or as food contact materials (FCMs) [44,45,46]. 85%–90% of the wall polysaccharides consist of a mixture of water-soluble mannans, soluble glucans (10–48%) and alkali-insoluble (15–48%), as well as minor amounts of chitin [47]. Contents may vary, generally accepted being 40% mannoproteins and 60% β-glucans [48] and, depending on the analysis, the cell wall can represent between 15% to 30% of the dry mass of the yeast cell and its thickness is from 105 nm to 200 nm [41,49].

Beta-glucan synthesis takes place in several cell regions. The process of glucan formation occurs in the plasma membrane and is catalyzed enzymatically by glucan synthase (UDP-glucose; 1,3-β-D-glucan 3-β-D-glucosyltransferase, EC 2.4.1.3) encoded by the FKS gene (1 and 2), which, by the extrusion, incorporates newly formed chains in the cell wall [50]. The order in which these compounds are found in the cell wall is: β-1,3-glucan → β-1,6-glucan → mannoproteins [51]. The degree of polymerization of β-1,3-glucan depends largely on external factors and is achieved through two synthase pathways related to the growth phase and the carbon source [35]. Biochemical research on *S. cerevisiae* has indicated that the synthase bound to the cell membrane uses UDP-glucose as a substrate and consists of a cytosolic regulatory subunit and a catalytic subunit with an affinity for ATP and GTP [52,53].

Cell wall mannoproteins are indirectly connected by β-1,6-glucan through a glycosylphosphatidylinositol (GPI) anchor and directly by β-1,3-glucan through bonds that break relatively easily in alkalis [54]. This layer, with a thickness of 30–40 nm, is located toward the cell surface [55] has a protein fragment of approximately 100 kDa that can be partially removed chemically while the rest requires the use of enzymes [56]. Although this amorphous layer can be removed by alkaline or enzymatic treatment for a high purification of β-1,3/β-1,6-glucan, some studies indicate that the glucan-mannan complex is also suitable for food or pharmaceutical purposes [57,58].

**Figure 1:**
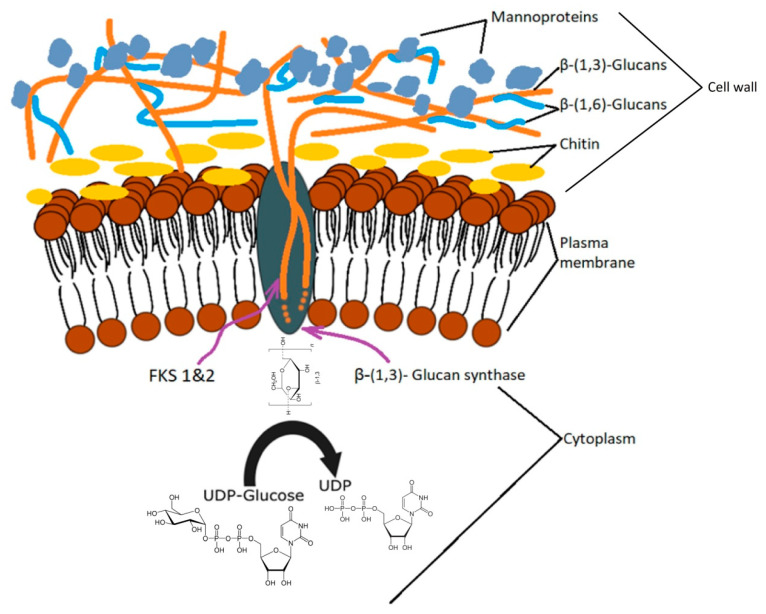
Schematic representation of the yeast cell wall and distribution of β-glucans in *Saccharomices cerevisiae* (adapted from [42,59]).

A schematic representation of the yeast cell wall adapted from Klis et al. [42] and Cabib et al. [59] is illustrated in Figure 1.

### 2.3. Serial Repitching and the Thickness of the Cell Wall

During serial repitching, the morphology of the yeast cell undergoes changes from smooth to wrinkled [33]. In the brewing process, serial repitching of yeast biomass is usually used between four to six times before its disposal [60] but can be employed up to 15 times. During a fermentation cycle the cells can divide two to three times resulting in about 13–25 replications [31]. As a consequence, it results in a genetic drift that develops stability to the environment in which grows. These changes reach values of 10–25% of the total cell mass and can be easily seen through atomic force microscopy (which is a useful tool in the analysis of cell walls that undergo genetic mutations during fermentation) [61]. Thus, cell rupture by methods such as enzymatic hydrolysis described in the literature becomes difficult at up to 3.5 times to obtain the same degree of hydrolysis compared to unrepitched yeast, most likely due to the thickness of the cell wall, that increases in serial replications [62], and to stability from the growth media.

A study performed over a period of 20 days, indicated that some mutations in yeast strains during cell autolysis, that occurs at the end of the stationary growth phase, led to a decrease in β-glucan content (from 119.23 µg/mg to 57.73 µg/mg) and a deposit in chitin content (10.98 µg/mg) due to the destruction of the FKS1 gene that synthesizes β-1,3-glucans [63]. Under the influence of the FKS1 and FKS2 genes, the shape of the cell changes from spherical to ovoid [55] and, after 10 days of autolysis, a 26.6% decrease in cellular dry weight can be observed in *S. cerevisiae* [64]. Any replication means a change in the wall and conditions the age of the cell [65]. Cell wall damage activates the so-called cell wall integrity pathway (CWI), which in terms of wall stiffness, in addition to the significant chitin deposit (at the buds and on the side walls), also involves synthesis of the β-1,6-glucan-chitin complex at its reducing ends and an increase in β-1,3-glucanase resistance to the growth condition, protecting the cell from degrading enzymes [42]. In the stationary phase of brewer’s yeast, the cell wall increase in thickness and changes that are produced in structure results in a 1.64-fold increase in β-glucan content [66].

## 3. Spent Brewer’s Yeast

According to research, yeast residues from brewing constitute between 1.7–2.3 g/L of beer [67] or up to 1.5–3 kg/100 L of beer [68], which are recovered through the sedimentation process [20]. The main destinations are animal feed or yeast autolysates for human consumption due to the high content of antioxidant compounds (up to 32.73 mMol TEAC/100 mL) and amino acids [69,70]. Yeast residues represent up to 15% of the total by-products from brewing processes [71] and have a content of about 40% crude protein, 59% total carbohydrates (from which 23% are β-glucans) and 1% lipids [72].

In 2004, Desai et al. [73] predicted an up to 0.077 g/L yield of β-glucan by using a neural network and growth media extract. Thus, the yeast cell wall architecture depends on nutrient availability. Due to the growth conditions during brewing, it can be speculated that there is a certain heterogeneity in the glucan content so that the response of the immune system turns out to be different from that induced by other glucan forms such as from baker yeast [74]. The products extracted from the yeast cell wall sold under the name Yestimun or Wellmune are marketed for the immune strengthening system to reduce the susceptibility to common colds during the cold season [75]. The culture media has a special influence on the antioxidant activity of the extracted β-glucans. Jaehrig et al. observed that the use of wort growth media increases antioxidant activity in yeast cells [76].

### 3.1. Characterization

Spent brewer’s yeast has found few uses in the food industry [43], especially due to the bitterness attributed to hop resins during fermentation [77]. The process for removing bitter compounds is easily achieved by methods such as rotary microfiltration or alkaline treatment [77,78]. The resulting compound is then used in the formulation of functional foods such as yeast extracts or beta-glucans [79].

It has been reported that the cell wall structure of *S. cerevisiae* undergoes visible changes during the fermentation process of high gravity brewing [66]. Yeast strains develop specific mutations allowing better use of sugars from media (maltose in lager yeast or a high affinity to the fructose transporter in wine yeast with the same species *cerevisiae*) [22]. Other studies, such as those of Aguilar-Uscanga and Francois, observed in four independent experiments an increase in polysaccharide content (β-glucans) from 14% to 21% in cells cultivated on alcohol [80]. Thus, apart from the adaptive response to stress, which depends on the metabolic conditions of cells [60,81] during the fermentation, the carbon source from the media influences the polysaccharide composition of the cell wall.

Such changes during fermentation were highlighted by an ultrafine section of yeast samples with a diamond knife and examined using a transmission electron microscope (TEM), in which it was clearly observed that the thickness of the cell walls after replication increased [33]. Thus, the cell walls of the spent brewer’s yeast reached 225 nm in thickness (data are taken from the images of Tian et al.) [82] and were 25 nm thicker than the average described in the literature. Furthermore, with an increase in glucan content of up to 21% (predominantly of the short β-1,6 fraction linked to β-1,3 glucan in cells grown in ethanol), the resistance to lytic action of zymolyase, and the mechanical strength to cell rupture, grew [80].

### 3.2. Bitter Compounds and the Possibility of Their Removal

Compared to fresh yeast, the resulting yeast from the fermentation processes must be subjected to a pretreatment to remove bitter compounds to have a food grade. The compounds that give the yeast a bitter taste originate from hop resins [83]. They are adsorbed on the yeast during fermentation in a percentage of 9–27% of the total bitter substances from wort [84] and are mainly represented by humulons and isohumulons [5]. The debittering process is mainly used to obtain yeast extracts that have a high nutritional value [60,70] and less for β-glucans because bitter substances are considered to be removed by washing or during extraction [72,82,85]. The only debittering process described for β-glucans is performed at pH = 10 and a temperature of 50 °C within 10 to 30 min followed by centrifugation and washing [86,87].

These particular compounds of brewer’s yeast can be removed without affecting the cell, whereas bitter compounds are found on the surface and less in the cytoplasmic content. In order to eliminate the hydrogen bonds formed by adsorption forces, and to preserve the cellular integrity, Simard and Bouksaim managed to remove up to 98% of the bitter taste after 5 min. This was achieved by treating a yeast suspension with 2 N NaOH, pH = 10 at a temperature of 50 °C, and the addition of 20% polysorbate could completely reactivate the cells [88]. Measured in bitterness units (BU), Kim, 2001 observed that at a pH of 10.5 after 3 min of contact, the yeast suspension no longer showed a significant change in bitter taste (from 25.2 BU to 23.1 BU after 30 min), while at an increase in pH to 12, bitterness reach values of 3 BU after 30 min. Of course, this last aggressive process after 10 min reduces the viability of cells to zero. Optimal parameters for a mild debittering with a cell viability of 88% and 24.1 BU would be: pH = 10.5/10 min, with a yeast suspension treated with 0.1% [89].

Another debittering process, without the use of chemical solvents that could be used to remove hop resins, is microfiltration. Rotational microfiltration at pH 5.5 that gives rise to the Taylor vortex leads to a considerable reduction in bitter compounds [78].

### 3.3. Glucan Content

Architecturally, the cell wall ranges between 15–30% of the dry mass of the yeast that has in its composition 50% β-1,3-glucans and 10% β-1,6-glucans [17]. With a simple calculation this means that yeast *S. cerevisiae* is theoretically composed of a mixed-linkage of β-1,3/1,6-glucans between 9% and 18% that can be isolated by various methods for further uses.

To maintain viability and efficiency, yeasts through the ability to respond to stress, increase their total glucan storage by up to 49% during beer fermentation [66]. The β-glucan content also increases with the maturation process when cold fermentation induces stress on the yeast cell [20]. These changes mainly depend on the media in which the yeast ferments and with the cell age (which is determined by the number of bud scars from serial repitching) [31]. Thus, polysaccharides can vary by more than 50% due to the growth conditions and reach values of 127.4 µg/mg in total β-glucans of the cell mass (meaning 12.74%) on YPD media (yeast extract, peptone, dextrose), and up to 21% under stress on ethanol cultivation [80]. These results are also supported by the Wrobel et al. research, which through the cultivation of a brewer’s yeast strain (*S. cerevisiae* R9) on different culture media managed to obtain a total β-glucan content of up to 20.2 g/100 g of dry mass (representing 30.6% of the total carbohydrates); the rest were soluble fractions of β-glucans (16%) and mannoproteins (26.4%) [90].

## 4. Beta-Glucans

Yeast cells are continuously studied for the discovery of new models and polysaccharide structures with therapeutic uses, or as ingredients in functional foods. To understand the true structure-function relationship of yeast glucan complexity, research refers only to the β-1,3/β-1,6-glucan bonds. Since the beginning of 1894, when the chemical composition of the cell wall was studied to analyze residual polysaccharides after digestion with NaOH [26], until now when the particulate β-glucans from the cell surface by their three-dimensional structure have immunopharmacological activity [91], β-glucans have found multipurpose uses such as adjuvants in vaccines (due to a robust stimulation of CD40/CD86 antigens) [92], hydrogels in the administration of vitamin B12 (consisting of a chitin-glucan complex) and for their antioxidant, antibacterial and anti-inflammatory effect [92,93].

β-1,3/β-1,6-glucans present different conformations such as side chain branches, branch on branch or cyclic molecules. These conformations consist mainly of D-glucose units which are linked by β-glycosidic bonds [94]. They vary in the type of anomeric configuration of β-d-Glcp units, the spatial distribution of glycosidic bonds, degree of branching, the degree of polymerization and molecular weight. Hence, we can distinguish linear, branched or cyclic β-glucans with different glycosidic bonds, which are influenced by the fungal species [15]. Highly branched β-1,3-glucans are thus shown to have a much higher immune-stimulating ability than linear shapes found in bacteria [95].

The cell wall glucans are classified into two different categories due to solubility in alkaline substances. They are 80–85% insoluble in hot alkaline substances (75 °C, 0.75 M solution) because of covalent association with chitin and other polysaccharides and soluble in water [76,94]. The study of this insoluble property is the aim of this research. In contrast to plant cell walls, that have as their main polysaccharide cellulose consisting of a linear chain of β-1,3-glucan interconnected with β-1,4-glucan [96], yeast glucan forms are composed of glucose monomers having a basic structure of β-1,3 interconnected in the side by β-1,6 chains [97]. The primary structure covalently binds to other components of the cell wall so that the nonreducing ends are cross-linked by β-1,3, β-1,4 and β-1,6 bonds to the reducing ends of chitin [98]. This is explained by the insolubility of β-glucans in alkaline substances and, due to the intermittent connections, a three-dimensional β-1,3/β-1,6 network is formed [76,99].

The degree of branching (DB) and polymerization (DPn) of insoluble β-1,3/β-1,6 glucans are 0.003 and 228, respectively [100]. So, β-1,3-glucan constitutes about 50% of the dry mass of yeast cell wall and has a degree of polymerization of 1500 glucose units, while β-1,6-glucan has 140 glucose units [101,102]. Another integral part of β-glucan cell wall is glycogen (α-1,4/1,6-D-glucan) which is separated by solubilization after acetic acid treatment or degradation by glucanases [15]. The fiber length of β-1,3-glucan reaches about three to six times the wall thickness [94].

**Figure 2:**
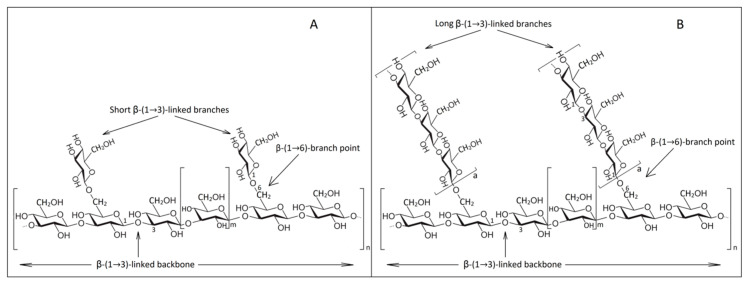
Schematic representation of yeast β-glucans: (**A**) soluble β-glucans; (**B**) insoluble β-glucans (adapted from [103,104]).

A schematic representation of the molecular distribution in β-glucans after Waszkiewicz-Robak [103] and Chan et al. [104] is described in Figure 2.

### 4.1. Chemical Composition and Food Safety

The US Food and Drug Administration classified yeast β-glucans generally recognized as safe (GRAS) and can be used up to 3 g/day [105]. A scientific opinion on the marketing of yeast β-glucans in the European Union was drawn up in 2011 by the European Food Safety Authority (EFSA) which evaluated β-glucans to be a safe product that can be used both as soluble and insoluble part in food supplements in the proportion of 375 mg/day or up to 600 mg/day for special food purposes [106]. This decision was completed by the European Union Directive from 2017 and revised in 2019 in which the use of β-glucans from yeast *S. cerevisiae* was extended to other foods such as juice drinks (1.3 g/kg), cereals for breakfast (15.3 g/kg), biscuits (6.7 g/kg), powdered milk (25.5 g/kg) and up to 3.8 g/kg in dairy products [107]. This type of polysaccharide, with a molecular weight of 100–200 kDa, extracted from the yeast wall is considered a novel food ingredient highly purified, consisting of β-1,3 and β-1,6 glucanic structures to which are attached chitin and various mannoproteins. The degree of purity of this water insoluble compound but dispersible in liquid matrices, must be above 80%, having the following specifications: protein < 4%; fat < 3%; ash < 2% and humidity < 6% [108]. Maximum limits of heavy metals allowed are lead < 0.2 mg/kg; arsenic < 0.2 mg/kg; cadmium < 0.1 mg/kg and mercury < 0.1 mg/kg [108].

### 4.2. Solubility

With respect to bioactive function, special attention is paid to the water-insoluble native form of yeast β-glucans [109]. The insolubility in water, alcohols or organic solvents of yeast β-glucans is mainly due to the intermolecular complex between chitin and the polymer chains located in the β-1,3 position that coexist in a percentage of 1% of the cell wall mass [103], but also due to the strong hydrogen bonds formed between the hydroxyl groups of glucose in the polymer chain. Another major constituent that confers rigidity are mannoproteins found on the outside of the cell wall, which are bound to the β-1,3/β-1,6 chains of β-glucan through covalent bonds [54], and a degree of polymerization greater than 100 of β-1,3-glucan makes it completely insoluble in water [103].

During extraction, some of the insoluble glucans pass into soluble form due to various methods applied in cell wall fractionation (extraction with NaOH/concentrated acids or by using DMSO/H_3_PO_4_) [110]. Their solubility increases as the degree of branching decreases [111]. A degree of polymerization less than 20 of the β-1,3/β-1,6 complex leads to a weakening in the molecular interactions and forms water-soluble compounds [112].

Both preparations can find medical applicability. Various soluble forms of orally administered β-glucan protect against *S. aureus* or *C. albicans* infections and can stimulate the innate immune system through specific proteins (PRRs) [113]. In contrast, the insoluble form from yeast is desirable because it passes unchanged through the gastrointestinal tract and activates specific β-glucan receptors [111]. It has an increased affinity to dectin-1, a receptor for β-1,3/β-1,6 chains that induces an avalanche in the innate and adaptive immune response [74]. A simple experimental study conducted with a number of 42 different glucans showed that the β-glucans from yeast have the strongest stimulating effect on the immune response [114]. Such compounds can be easily incorporated into various matrices for oral administration as medicines or in the food industry, demonstrating their efficiency in common colds (only 47 people became infected in the β-glucan group compared to 72 in the placebo within 13 weeks of study) [72,115,116].

## 5. Extraction Methods

Insoluble β-glucans resulting from extraction come into two forms, which are explicitly described in the literature. These are whole β-glucans particles that retain the spherical shape of the cell, are hollow inside, and are successfully used as an encapsulating agent for various drugs [117,118], or fragments of β-glucans from the cell wall obtained by mechanical methods of cell rupture [119,120]. This last process disintegrates the cell allowing faster access to contact of the solvents with the intracellular content to be removed. Due to the rigidity and thickness of the wall, yeast cell *S. cerevisiae* is resistant to lytic action and some singular processes are not very efficient to break the cell wall. Therefore, in practice, combined methods are used such as heat treatment combined with ultrasound, which in addition to a decrease in cellular rigidity, also leads to the removal of amorphous impurities found between β-glucan fibrils [121].

From alkaline treatments to mild combinations with hot water at 125 °C and organic solvents [86,122], various methods are used to isolate and to purify β-glucans from yeast so that the three-dimensional structure (described by the simple curdlan model) is not affected, and if this occurs, the recovery of random transitions of the polymer chain can be achieved by drying at 55–60 °C [123].

Large glucan aggregates have up to eight triple spirals arranged in the form of rigid rods [124]. Due to external mannoprotein structures, certain protection is conferred against the treatments that remove cellular components after extraction, resulting in whole, spherical, hollow inside particles made up of β-glucans that keep the same dimensions as the original cell [125]. In fact, even after treatment with NaOH used separately (followed or not by ultrasound for the removal of β-glucan conglomerates), it is observed that the glucan extract retains the spherical shape as the yeast cell [86].

An analysis of glucan content and discrimination from other components such as α-glucans is done using rapid measurement kits by methods based on acid and enzymatic treatments to hydrolyze β-glucan. For a more detailed characterization of the structure and conformation, along with microscopy, useful are nuclear magnetic resonance spectroscopy and Fourier-transform infrared spectroscopy [100,126]. In the following, a number of convenient methods that can be done in the extraction of β-glucans from spent brewer’s yeast are highlighted.

### 5.1. Initial Purification

For food grade, spent brewer’s yeast must be subjected to successive steps of initial purification. Many of the β-glucan extractions are made from fresh yeast so it would be acceptable to bring the residual yeast to a level close to that in which the extraction methods are well defined by the literature. Therefore, it is necessary that yeast slurry is first centrifuged to remove beer liquor, as authors such as Tanguler and Ertenand Vieira et al. did in obtaining yeast extracts [5,127]. Then the obtained paste needs to be resuspended in water and meshed through micrometric sieves to remove the components of the residual wort as well as wheat bran [82]. Parameters such as sieve size, dry matter of yeast slurry, speed of centrifugation or number of washes used at each stage vary depending on the method, and must be optimized in the extraction of β-glucans.

It is necessary to purify over bitter substances from hops that are present in beer as solids or adsorbed on the surface of the yeast cell [5]. It is tempting to use, in removing the bitter compounds, the fastest possible method described above pH 12 that reduces the viability of yeast to zero, or to skip this process because the next steps will require aggressive combinations of compounds to release intracellular content and implicitly will lead to wall fragmentation and debittering. This is controversial because many researchers predominantly use the same treatments for fresh or cultivated yeast on selective media [9,10] regardless of the fact that any reaction other than standard treatment has implications for the method and will reduce the efficiency of β-glucan extraction (for example in NaOH extraction at a ratio of 1:5, the five volumes of alkali will form complexes with bitter substances if they have not been previously removed). Second, changes in cell wall architecture during fermentation and after autolysis [55], the number of bud scars after serial repitchings [32] or the formation of natural hybrids [128], might be additional obstacles that makes it difficult to break or release the intracellular content from spent yeast.

Moreover, further aggressive treatment can affect degrading β-glucan chains [76] and thus the reliability of the bioactive effect described in the literature is not as expected. It is, therefore, desirable that debittering of the spent brewer’s yeast is a distinct part of the extraction process and to be conducted before cell lysis. Thus, we find it opportune to use the process described by Simard and Bouksaim in which bringing a yeast suspension to a pH of 10 with 2 N NaOH at a temperature of 50 °C, the bitter aroma is removed in a percentage of 98% [88]. Any other process that does not severely affect the integrity of the cell other than to break the hydrogen bonds formed by adsorption with hop resins is appropriate. The purification of compounds from the residual yeast being the subject of the further research.

### 5.2. Cell Lysis and the Breakage of the Cell Wall

Currently, a number of methods to improve the process of cell lysis to allow the release of cytoplasmic contents exists. From the chemical, physical or enzymatic among the most accessible methods in the extraction of glucans are milling with glass beads in diameters of 0.25–1 mm [26,57], induced autolysis at 55 °C [120], cell rupture with enzymes such as *Actinomyces rutgersensis* 88 or zymolyase [129,130], homogenization at high pressures or ultrasound [72,131], alkaline treatment at 60 °C [132], pulsed electric field (20 V and 100 µs to not cause electrolysis) [133], enzymatic combinations and ultrasonic methods [131].

*Saccharomyces cerevisiae* cells are very resistant, and due to the thickness and rigidity of the cell wall some simple methods such as ultrasound are not enough for cell death [134]. Each method has its advantages, so we must consider whether to obtain fragments or whole-spherical particles of β-glucans because cell lysis with glass beads or a strong chemical treatment will cause cell rupture at the beginning. A detailed description of yeast cell-breaking strategies, energy consumption and the efficiency of cell rupture techniques is described by Liu et al. in a review that highlights the bioactive compounds of *S. cerevisiae* [40]. Another six simple methods of cell rupture, including osmotic shock or freezing/defreezing, are presented in research by Kot et al. for extracting cellular lipids which are suitable in the extraction of β-glucans [135].

#### 5.2.1. Autolysis

One of the most commonly used methods that cause the death of a yeast cell death is the process of cell destruction by its own enzymatic mechanisms. Autolysis is a form of enzymatically cellular death that takes place under the action of its own enzymes, in which the cell wall is partially destroyed allowing the cytoplasm to evacuate outside the cell. Defined as the hydrolysis of intracellular polymers under the action of hydrolytic enzymes associated with cell death [129], induced autolysis occurs at temperatures between 50–55 °C within several hours [120,136], or it may be due to senescence after serial replications during fermentation [137].

Autophagy is a long process and starts in lysosomes where digestive enzymes are released into the cytoplasm [129]. Wang and colleagues analyzed the process of autolysis during beer fermentation, concluding that there are three distinct phases, namely: the stationary phase, anaphase and the autolysis stage. In these three stages, cells undergo transformations from full and intact to slightly erase (after a month in fermentation liquor) so that finally, due to degrading enzymes, cytoplasmic content is completely released through holes formed in the cell membrane [55].

A percentage in this autolyzed yeast is found at the initial reception, the rest requires induction of lytic process by increasing the temperature. Thus, for autolysis the extraction of β-glucans from yeast suspensions that have been brought to a certain solid substance content (generally 15% *w*/*w*) and a pH of 5, 3% NaCl is added to initiate the lytic process. The heat treatment applied under continuous stirring is 24 h at 55 °C [120]. Parameters such as temperature and time vary depending on the method and solid content, so Zechner-Krpan et al. after pretreatment by meshing and debittering with NaOH, left the yeast for 36 h at 50 °C to induce cell death [86]. Optimization of these parameters for obtaining extracts from spent brewer’s yeast was also studied by Tanguler and Ertenin at a range of temperatures between 45–60 °C and a reaction time between 8–72 h. They concluded that 45–50 °C and 24 h are adequate for releasing the main intracellular solids in a proportion of up to 64.1% [5].

At the end of autolysis for the inactivation of endolytic enzymes, the yeast autolysate is pasteurized by heating at 80 °C 15–30 min, cooled and centrifuged so that it can be used in the next extraction steps [5,120]. The autolysis ratio is calculated as the total loss of dry biomass during the lytic process.

#### 5.2.2. Enzymatic Treatment

Various enzymes can be used to speed up cell death. These include zymolyase, peptidase, glucanase, lipase, lysozyme or glycosidase that breaks down cellular structures [40].

Cell rupture first occurs under the action of protease and glucanase that attack the surface of the cell wall. Choosing of glucanases should be proportionate so as not to damage the main structure of β-glucans but only to allow the release of the intracellular content. This may be the case when the enzyme complex *Actinomyces rutgersensis* 88 is used successfully in the extraction of β-glucans with established β-glucanase activity, with an optimum of 1.5 units (40 mL enzyme suspension), 6 h/50 °C and pH = 10 [129]. Another approach, with the help of enzymes for cell wall rupture and β-glucan extraction, is treatment with Glucanex^®^ 200G developed by Varelas et al. that applied three enzymatic treatments on a strain of *S. cerevisiae* at different enzyme concentrations. They concluded that optimal β-glucan content is obtained using a ten times normal enzyme concentration (10 × 0.015 g/L) [101]. Other enzymatic combinations on spent brewer’s yeast to produce a food grade yeast involve a concomitant treatment with endoproteases, exoproteases and deaminases that end up obtaining a solids content of up to 55.1% [138].

#### 5.2.3. Cell Disruption with Beads

In addition to homogenization or ultrasound, cell disruption methods with the use of glass or zirconium beads are a fast and convenient mechanical method in the recovery of cellular components. Cell rupture, a complex process that is largely dictated by the size of beads and the concentration of yeast suspension is divided into two stages: breaking the cell wall and releasing the intracellular contents. Circular beads, in addition to breaking the cell walls, also act as a homogenizing factor, so that some of the enzymes released in the continuous phase interact with the cellular structures giving rise to a chained lytic process.

In addition to cell rupture, the size of beads has a direct impact on the enzymes located in different parts of the yeast cell. A smaller diameter of beads yields a higher enzymatic release [139]. Choosing the concentration of suspension is also essential at this stage because an increase in disintegration rate has the effect of a mutual blockage with the contact surface of the beads, resulting in insufficient collision with living cells [140]. For the cell breakage of spent brewer’s yeast to obtain β-glucans, beads with a diameter between 0.25–1 mm are used [57]. An optimum for fractionating yeast cells would be around 0.4–0.5 mm in diameter [28,121], and a too large a bead size (of Ø = 1 mm) causes the formation of cell aggregates that have a negative effect on the lytic process [119].

#### 5.2.4. High-Pressure Homogenization

Homogenization at high pressures is one of the common mechanical methods in cell inactivation and rupture. This treatment accelerates the lysis of yeast to a level desired to achieve various cellular components. To extract β-glucans, a treatment at 800 bar and a single pass through the homogenizer of a suspension of 9.2 logCFU/mL yeast proved to be sufficient for cell disintegration, reaching values of 0.87. The next two passes with values of 0.97 were not significant in the pre-extraction stages [141]. Delgado et al., in order to prepare *S. cerevisiae*-based films, managed to inactivate a 10% w/v suspension in a continuous flow of 9 min at pressures of 125 MPa (1250 bar) [142]. Other combined treatments for weakening the cell wall with hot water and then cell rupture by homogenization have found their applicability at lower pressures (70 MPa and three passes) reaching values of cells disintegration to 95.50% *w*/*w* [120].

**Figure 3:**
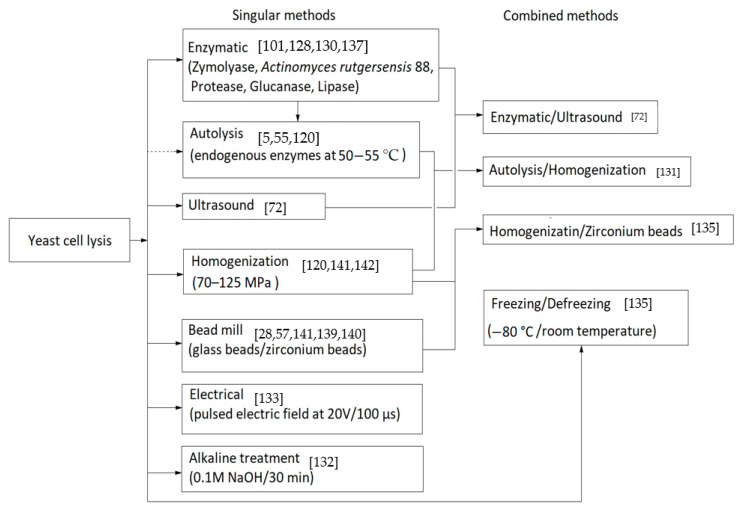
Different cell lysis methods for obtaining β-glucans.

Figure 3 shows some of the cell lysis methods used in β-glucans extraction from yeast cells.

### 5.3. The Proper Extraction

β-glucan extraction is performed after the end of the lytic process and continues to remove the intracellular contents and amorphous structures from the cell walls. Removal of intracellular contents and amorphous structures can be done with NaOH, H_2_O_2_ or acid solutions, and is based on the ability to dissolve polymers. High molecular weight polymers such as β-glucans can be insoluble in alkaline solutions at concentrations up to 10 M [129].

As a rule, different extractions, like alkaline, acidic, combined or innovative require successive centrifugation, which is necessary to separate the insoluble part. The number of centrifugations, time and speed are variable and differ with the method.

#### 5.3.1. Alkaline/Alkaline-Acid Extraction

Perhaps one of the most commonly used and safest extraction methods, with a long history behind it, is alkaline extraction (since 1969 Bacon and 1973 Manners investigated β-1,3 and β-1,6 structures insoluble indifferent NaOH concentrations) [110,143]. The extraction process is carried out in several stages and is based on the treatment at high temperatures (approximately 90 °C) with 1 N NaOH in a ratio of 1:5 by volume (one volume of cell suspension to five volumes of hydroxide) and then, after the removal of the supernatant (consisting of nucleic acids, proteins and lipids), an acid treatment (particularly acetic acid in the same proportion of volume) is used for glycogen removal and amorphous compounds [144,145]. Other possible combinations found in the literature are NaOH/HCl, NaOH/CH_3_COOH, NaOH/NaClO and NaOH + NaClO/DMSO. High quality of glucan extract from all these methods is described by the use of acetic acid [146].

Even if strong bases are recognized to degrade polymer chains and thus result in a lower extraction yield (from 8.4% to 10.4% compared to 11.2% in the method displayed by Liu et al.) [120], the reliability of alkaline-acid extraction is still recognized (2020) and is used to obtain particulate β-glucan used for drug encapsulation such as with ibuprofen [147]. Other approaches that demonstrate the importance of the alkaline-acid method by the proven immunoactive effect involve successive treatments with 3% NaOH and 1 N HCl [9]. For better dissolution of mannoproteins and a concentration in β-glucans, Javmen et al. aimed at the dependence of dissolved glucose over the variation of NaOH solution, finding an optimum of 0.5M four hours after treatment [129].

Regarding only the residual yeast, through a unique process with NaOH in 2010 Zechner-Krpan and colleagues managed to extract β-glucans at a yield of 7.33% [86]. The other three extracts examined by Petravic-Tominac, namely alkaline, alkaline-acid and alkaline-acid extraction with the removal of mannoproteins (with a yield of 7.33%, 11.84% and 8.08%, respectively [86,148]) have been successfully tested and promoted for possible food applications [149].

In 2019, Tian et al. in order to optimize alkaline extraction used the preliminary homogenization of the cell wall by two passes at high pressure of 1700 bar followed by holding in NaOH with glass beads that led to a yield of up to 9.78% β-glucans from residual cells [82].

#### 5.3.2. High-Pressure Homogenization

This technique is mainly used for inactivating microorganisms by mechanical destruction of cell walls at high pressures up to 250 MPa [150], but it can also be used in the extraction of β-glucans because the homogenized liquid phase becomes easier to remove from insoluble polysaccharide fragments. The mechanical resistance of yeast described above to be between 80–104 µN, can be successfully suppressed for destroying cell walls. Kleinig and Middelberg described that compressive forces of 150 µN are capable of destroying yeast cells of 6 µm in size and can be reached at pressures of 56 MPa using a high-pressure homogenizer [151].

Liu et al., to bypass alkaline extraction after a water treatment at 121 °C, described a new method of β-glucan extraction from spent brewer’s yeast through homogenization and other additional treatments (in addition to hot water treatment, a treatment with organic solvents and one with proteases are added), reaching a yield of 11.19% (*w*/*w*), a value that can be calculated from the article and is related to the initial solid by multiplying the yield in solids with the calculated β-glucan (13.33% × 84%) [120]. The optimum homogenization pressure chosen after tests in a range of 40–80 MPa was 70 MPa with three successive passes through the homogenizer (with a fragmentation ratio of 95.50%).

#### 5.3.3. Enzymatic Extraction

To avoid alkaline-acid methods considered to be degrading to β-glucans, some researchers introduced enzymatic extraction as an alternative to treatments with strong chemical solvents. Thus, if the enzymes used in the above-mentioned lytic process are left in the system, they lead to a cell rupture until glucan residues are obtained. The enzymes mainly used in the extraction and purification of β-glucans presented in the literature are proteases [120,152]. Concerning proteases, after a treatment to the cell walls for 5 h at pH = 10.5 and 45 °C followed by successive washes of the sediment with acetone or ethanol, maximum values of β-glucans of 26% and 25% are reached (with a purity yield of 85% and 83%, respectively) [152]. The combination of enzymes in the extraction process is also discussed so that proteases (Savinase) and lipases (Lipolases) that are used after hot water treatment (125 °C/5 h) are described as soft treatments [153].

It should be mentioned that in these two articles presented by Freimund et al. and Borchani et al., the extraction of β-glucans started from *S. cerevisiae* procured in the form of cell walls; in the last article a yield of 18% and a purity of the glucan content of 79% was achieved. Tam et al., by using Alcalase^®^ 2.4 LFG (another type of endopeptidase used for protein hydrolysis) and combining the enzymatic treatment with ultrasounds on a spent yeast suspension adjusted to 15% (*w*/*w*), reached a purity of 72.06% from the glucanic compound (no reference was made to the yield of β-glucans) [131].

#### 5.3.4. Other Extraction Types

Continuous research in this area makes us look for the most favorable methods in order to obtain glucanic compounds that have different properties on the body which are specially related to the length of the polymer chain, the degree of solubility or purity of the extract. Below are some of the methods described to be innovative that lead to the isolation of β-glucans from yeast.

Ionic liquids extraction is one of the new methods considered to produce β-glucans of high purity at high yield. Thus, Pham et al. managed to extract β-glucans with a purity of 95.2% at a yield of 83.5% (reported to the total glucan content from yeast) using 1-butyl-3-methylimidazolium chloride [Bmim]Cl. Due to the ionic nature of [Bmim]Cl, which has a high content of cations and anions, considerable polarity and a low melting point (extraction taking place at 80 °C), from 1 kg of dry yeast (having 14.3% β-glucan) 125g of β-glucan was obtained (thus 14.3 × 83.5 = 119.4g pure β-glucan) [154]. Their precipitation from solution was achieved by the addition of water followed by filtration or centrifugation.

However, with spectroscopic analysis of circular dichroism over the range of 183–250 nm, a negative peak is observed for untreated β-glucan at 185 nm, showing a gradual bathochromic effect with the application of ionic liquids up to 187.5 nm, which demonstrates a deterioration of the three-dimensional structure. Thus, the ionic treatments conducted in the isolation of β-glucans such as 1-ethyl-3-methylimidazolium acetate [EmimAc] and combined with homogenization at high pressures, can change the triple helix conformation to random chains that form new hydrogen bonds [155].

Combined extraction is perhaps the most common because it allows a better access to the cell wall with moderate use of materials and so the process becomes more convenient in the extraction of β-glucans. Such processes are used by combining materials such as alkaline and acid treatment, ultrasound with alkaline extraction [156], hot water/alkaline extraction, hot water/ultrasound and proteolysis, enzymatic/ultrasound and autoclavation [131,152,157] and alkaline-acid and enzymatic treatment [158].

Supercritical fluid extraction has become popular and is considered a green extraction method [159]. This can be achieved on a 10% suspension of autolyzed yeast by a treatment at 121 °C for 4 h [120]. Another method described by Jaehrig et al. involves an autoclavation treatment at 125 °C of a 13% (*w*/*w*) suspension within 5 h [76]. In each case, compared to the extraction of β-glucans from cereals, treatment with supercritical water and precipitation with organic antisolvents did not encounter difficulties such as the need for additional isolation due to the coextraction of starch [160].

**Figure 4:**
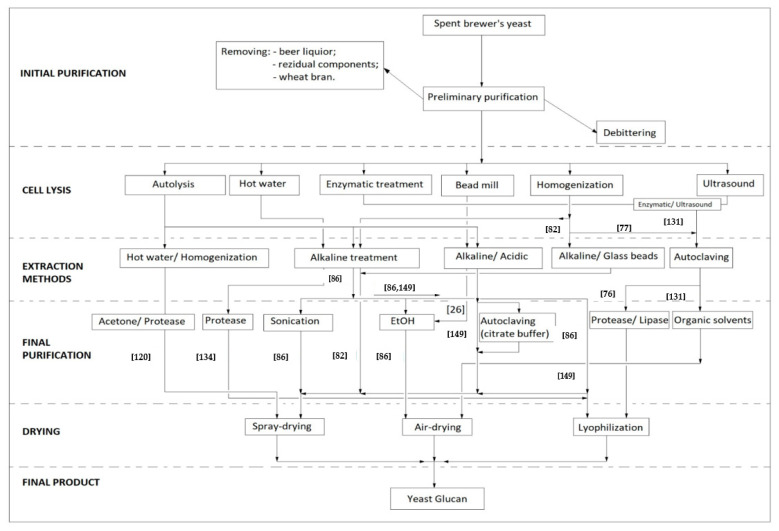
Protocols for obtaining β-glucans from spent brewer’s yeast.

Because a detailed description of the extraction methods which highlights each step cannot be done, the following diagram briefly sets out the protocol to be followed in order to obtain insoluble β-glucans from spent brewer’s yeast described in the literature. Below, Figure 4 shows different methods of obtaining β-glucans from spent brewer’s yeast.

### 5.4. Final Purification of Extracted β-Glucans

After proper extraction, in order to reach certain limits that meet actual requirements, insoluble β-glucans from yeast are subjected to various purification methods. In this respect, specifications such as those established by the Official Journal of the European Union in 2017 (CELEX number: 32017D2078; L295/78) which provide a content in β-1,3/1,6-glucans over 80%; proteins < 4%; fat < 3%; ash < 2%; humidity < 6% [161], or those previously published by the European Food Safety Authority in 2011 that evaluated β-glucans from *S. cerevisiae* as safe for human consumption (with a minimum of 75% insoluble glucan; proteins < 3.5%; fat < 10%; ash < 3%; humidity < 8%), must be considered [106]. The purification methods mainly concern the removal of protein and lipid content from the final extract and are described below.

Thus, Liepins et al. used the alkaline method repeatedly to purify β-glucans from spent brewer’s yeast. The working method involves a treatment with 3% NaOH 1:5 (*w*/*v*) initiated 4 h at 55 °C and then, after an overnight incubation at room temperature, the sediment formed was treated for 2 h at 100 °C in the same ratio of 1:5 with 3% NaOH. After this step the extracted sediment treated three times with 1 N HCl for 2 h was considered to be purified β-glucan [9].

To adjust protein content, it is known that alkaline treatment solubilized part of the cell wall mannoproteins [145]. Therefore, Suphantharika et al. optimized the alkaline extraction process so that the protein content decreased to 1.8% with an increase in NaOH concentration [162]. On the other hand, the alkaline-acid treatment of spent brewer’s yeast leads to the production of β-glucans with a protein content between 3.90% and 5.52% [148] and the use of hot water on the cell walls followed by homogenization at high pressure results in 4.30% protein, 2.68% lipids and 1.67% mannans [120]. Liu et al., by maintaining extraction with isopropyl alcohol and Protamex, managed to decrease the level to 2.99% protein, traces of lipids and 0.29% mannan [120]. By combining the two methods (alkaline treatment with high pressure), Tian et al. managed to reach a final purity of β-glucan of 78.11% [82]. Table 1 shows the chemical composition of β-glucans from brewer’s yeast purified by different methods.

**Figure 5:**
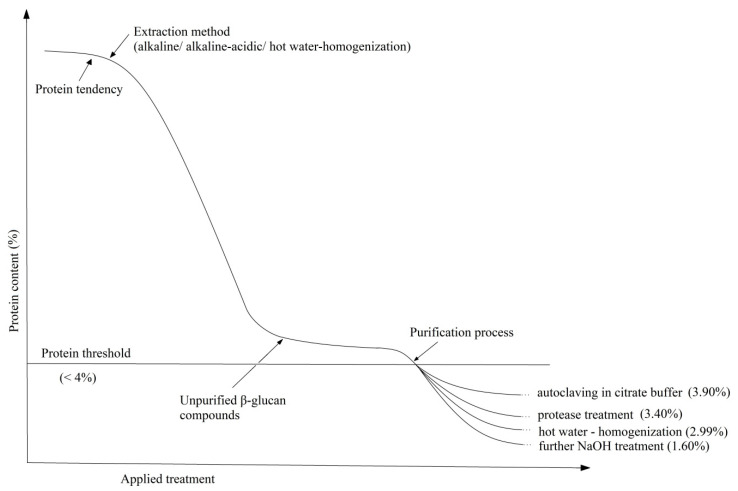
Schematic representation of protein content during β-glucan extraction.

As can be seen, the protein content decreases drastically with the alkaline treatment applied to the cell walls (from 43.47% to 6.54%) but not enough to comply with the limits of legislation requirements (<4%). Therefore, an optimum in the extraction process can be achieved by autoclavation and removal of mannoproteins, enzymatic treatment with protease, homogenization at high pressure or continuing the alkaline extraction. The last process with such low protein content can also influence the glucan chain leading to its deterioration. In fact, it can be seen in the glucan content of only 50.50%. The tendency of the protein composition as a result of the applied extractive processes can be easily followed in the schematic representation from Figure 5.

Further purification for various pharmaceutical purposes involving the fractionation of β-1,3/1,6-glucan into β-1,3 or 1,6-glucan are distinct parts of the extraction and are not detailed in this article. A documentation of these polymeric subgroups can be found in studies of Manners et al. in which enzymes such as β-1,6-glucanase and β-1,3-glucanase are used for the analysis of glycosidic bonds [110,163].

### 5.5. Drying

From the native model of curdlan (a simple unbranched β-1,3-glucan), which after dissociation in hot water can form reversible gels and by drying at 55–60 °C maintain three-dimensional structure [123], to complex processes such as ultrasound and spraying or lyophilization, each with different rheological properties, drying is reported to have remarkable implications on glucanic compounds [149]. Hromadkova et al., by examining the methods of drying β-glucans particles, extracted from *S. cerevisiae* by alkaline-acid process (NaOH/H_3_PO_4_) on immunoactive properties, concluded that spray-dried β-glucans show a significantly higher response on the immune system than those dried by lyophilization or after washing with ethanol and air-dried [164]. Another article published in 2015 by Liepins et al., with strict mention of β-glucans obtained from spent brewer’s yeast, supported that drying has implications in the immune response on peritoneal macrophages. Thus, desiccation before extraction combined with carboxymethylation increases the number of cytokines (TNF-α) by activating macrophages. β-glucans obtained from the yeast initially dried showed an FT-IR spectrum closer to the standard than those from which were not dried [9].

Rheological behavior of dry samples by various methods differs significantly. In fact, microscopic analysis can observe changes in the final compounds. Particulate β-glucan obtained by spray-drying retain the shape of the yeast cell (2–10 µm), being elliptical and compact with a smooth surface (implying that the spatial conformation of β-glucans remains preserved), while through the lyophilization, frozen water is removed from β-glucans by sublimation, resulting in deformed and compressed particles in the form of layers. Treatment with ethanol washing and drying in air leads to the formation of glucanic aggregates ten times larger in size than those that have retained the shape of the yeast cells. Authors concluded that if aqueous β-glucan suspensions are desired, it is recommended that the samples to be spray-dried [164].

A detailed description on the influence of drying methods on water retention or oil-binding capacity is presented in research by Petravic-Tominac et al. in which β-glucans isolated from spent brewer’s yeast and analyzed from the perspective of drying states showed that lyophilized samples had up to five times the ability of retaining oil than spray-dried and can be used successfully in the food industry [149].

## 6. Food Applications

Insoluble β-glucans from yeast are valuable functional polysaccharides used mainly for beneficial effects on human health because they stimulate the immune system, prevent infections and can be easily incorporated in sustained drug release [111], or can be used in the preparation of films [18]. The multidirectional role in bioactive properties has made β-glucans attractive ingredients for functional foods due to their prebiotic, antioxidant and blood lipid-regulation effects [71]. Besides its role in nutrition and health, many researchers use β-glucans for their stabilizing capacity in products such as soups, sauces and beverages [165]. Thus, they are introduced in food matrices as thickening agents, for water/oil binding capacity or as emulsifiers [72], to replace fat in mayonnaise [10] or for their pasting properties on wheat flour and starch [166]. There are plenty of possibilities for the use of β-glucans from *S. cerevisiae* in the food industry, thus Table 2 shows only those that refer to the β-glucans from spent brewer’s yeast.

### 6.1. Yogurt

Particularly, important in yogurt formulation with β-glucan is that it has the prebiotic role of these insoluble polysaccharides. The symbiotic role improves survival and effectively introduces live bacteria into the gastrointestinal tract [178,179]. da Silva Guedes et al. proved the cryoprotective role of β-glucan from spent yeast on lactic cultures during freeze-drying, refrigeration or exposure to simulated gastrointestinal conditions. They used three different lactic cultures and observed a decrease in bacterial cell membrane damage by up to 75.5% during freeze-drying, and the reduction in cell viability was only 1.2 log CFU/mL after 120 days of refrigeration due to β-glucans [173]. Raikos et al. determined that the physico-chemical and textural properties are favorable up to a level of 0.8% β-glucan. Skimmed yogurt fortified with polysaccharides reached the final fermentation pH of 4.5 one hour faster [175]. A study of the addition in nonfat yogurts of β-glucans up to a level of 2% concluded that at a concentration of 1.5% β-glucan, an important improvement in consistency, viscosity index and rheological properties during 28 days of storage was observed [144]. Also, Dos Santos et al. in 2019 by using β-glucan extracted through the alkaline method in concentrations up to 1% (*w*/*v*) noted major textural changes skimmed yogurt [176].

### 6.2. Mayonnaise

Another segment where β-glucans from spent brewer’s yeast are incorporated is mayonnaise. Enriching mayonnaise using different concentrations of β-glucans also aims to reduce fat content without influencing the content and physico-chemical properties of the final product. Thus, in 2006 Worrasinchai and colleagues partially replaced mayonnaise with β-glucans extracted through the alkaline-acid method up to a percentage of 75% showing that the viscoelastic properties were improved and an addition of insoluble polysaccharides not only replaced fats but effectively stabilized oil-in-water emulsions. A reduction of soybean oil up to 50% by adding β-glucans from spent brewer’s yeast was found to be acceptable [10]. After the fractionation of mannoproteins and β-glucans, da Silva Araujo et al. found their use advantageous in exhibiting special emulsifying and stabilizing properties during refrigeration [157]. Hence, many studies use β-glucans in fat substitution of mayonnaise preparation. The only changes observed in color can be masked by the addition of β-carotene or lutein [167,168].

### 6.3. Bread

Bread is also a subject of study, not only due to the fact that β-glucans act as dietary fiber but also for the implications they have on the glycemic control in diabetes by reducing blood sugar and insulinemia [116,180,181]. With a fiber content of 72.31%, β-glucans extracted through the alkaline-acid method and incorporated up to 2.02% resulting in an increased volume and a uniform distribution of pores in bread [171]. Extensive studies show that β-glucans have an influence from the dough stage. Thus, the highest strength, adhesion and stickiness are observed with the addition of 0.75% β-glucans from spent brewer’s yeast [172]. Furthermore, at this concentration, starch retrogradation after 4 days of refrigeration has the lowest value. This has also been observed with rice starch. Responsible for this effect are β-glucans which increase the thickening capacity and water retention [169].

### 6.4. Films

There is a growing interest in creating intelligent packaging made of biodegradable polymers as an alternative to synthetic materials that pose a health risk [182]. β-glucans can be easily modeled in development of films that keep the mechanical properties (tensile strength, elastic modulus and hardness) of synthetic packaging. Thus, Novak et al. developed a film based on β-glucan from *S. cerevisiae*, having as a plasticizer glycerol in a proportion of 25%. The elastic modulus, strength and tensile strength of these films were 712 MPa, 17.48 MPa and 14.16 MPa, respectively [18]. These are comparable to chitosan-based films at the same glycerol concentration (tensile strength of 17.97 MPa) [183]. Moreover, β-glucan-based films can also have a protective effect against molds. Sun et al. managed to protect pears from *Penicillium expansum* by coating with a layer of this polysaccharide applied on the surface of the fruit [184]. In an investigation of different types of β-glucans, Peltzer et al. concluded that the structure β-1,3/1,6 found in yeast has the greatest applicability for development of films for food contact materials, as well as in medicine [185].

## 7. Conclusions

Considering data presented in this review we can conclude that in brewing the serial repitching and manufacturing processes continuously affect cell rigidity and can lead to a more difficult extraction of β-glucans. Therefore, additional treatments are needed in the isolation process and repeated cycles of cell breakage might be effective in cell wall disruption

In order to extract β-glucans, it is indicated that the processing of yeast, which was serially repatched, be carried out as soon as possible to avoid the long-time autolysis process, because it can damage the polymer chain in the cell wall. To these aspects, separation from the bitter and alcoholized beer liquor must be performed immediately for lowering resistance to the lytic action of zymolyase and the mechanical strength to rupture cells.

An initial debittering of yeast suspension as a precell lysis step, not to interfere with the extraction and not to involve a high consumption of resources, is considered useful.

The role of yeast life span and growth conditions in the β-glucan content from spent brewer’s yeast can be evaluated as important considering that the energy invested through biogenesis in the form of carbohydrates is capitalized in bioactive compounds.

For an optimum extraction of β-glucans, we must consider the initial purification of yeast slurry which is performed through sieving, debittering and multiple washes.

If drying has so many implications on the immunological activity (such as those described by Liepins et al. [9]), we wonder if spent yeast should be dried before the β-glucan extraction process or we can only use one of the drying methods after extraction as Zechner-Krpan et al. described [86].

A rapid method for cell lysis can be achieved with the help of enzymes, but we must consider when to stop the lytic process to not affect the conformation and length of β-glucans (which are three to six times longer than wall thickness). In fact, this observation is also valid for the other extractive methods illustrated above.

For mechanical disruption by bead milling, the diameter of beads is critical so an average of 0.5 µm for yeast cell is useful.

Each method has its own advantages. The extraction process must be chosen carefully so that if we refer to the final shape of the whole glucan particle, high concentrations in alkalis will dissolve the native-spherical shape of the yeast and β-glucans can no longer be used as an encapsulating agent.

To fulfill legislative requirements, we must comply with proper chemical composition (polysaccharide, proteins, lipids) described by the authorities.

## Figures and Tables

**Table 1:** Chemical composition of β-glucans extracted from residual brewer’s yeast by different methods.

Extraction Method	Purification Process	Total Carbohydrates (%)	β-Glucans (%)	Protein (%)	Mannans (%)	Lipids (%)	Ash (%)	Reference (%)
Alkaline-acid	no	65.23 (59.61) *	55.21 (23.59) *	6.54 (43.47) *	nd	6.54 (43.47) *	0.55 (10.31) *	[72]
Alkaline-acid	Removal of mannoproteins by autoclaving in buffer (citrate)	93.04	95.25	3.90	nd	nd	nd	[149]
Alkaline	no	91.73	92.00	5.17	nd	nd	nd	[86]
Alkaline	Optimization of alkaline process for solubilizing the protein content	91.8 (55.20) *	50.50 (17.20) *	1.6 (41.20) *	nd	nd	nd	[162]
High-pressure homogenization and alkaline	High-pressure homogenization	nd	78.11 (16.16) *	2.29 (51.89) *	0.62 (9.75) *	0.33 (3.21) *	nd	[82]
Hot water	Protease and lipase treatment (Savinase and Lipolase)	nd	76.00 (30.00) *	3.40 (32.2) *	nd	0.9 (5.7) *	nd	[76]
Hot water	High-pressure homogenization, acetone and protease (Protamex)	95.84 (35.09) *	93.12 (20.58) *	2.99 (59.67) *	0.29 (12.72) *	tr (4.90) *	3.87 (7.07) *	[120]

* Number in brackets are percentage in chemical composition of the spent brewer’s yeast from which β-glucan was extracted (dry basis); nd—not determined; tr—traces.

**Table 2:** The use of β-glucans from spent brewer’s yeast in food industry.

Properties	Extraction Method	Reference
Pasting properties on wheat flour and starch	Alkaline	[166]
Fat replacer in mayonnaise	Alkaline-acid	[10,167,168]
Thickening agent, water/oil binding capacity or as emulsifiers	Alkaline; Alkaline-acid; Alkaline-acid with mannoprotein removal	[72,149]
Gelling capacity and preventing rice starch retrogradation	Alkaline-acid	[169,170]
Improving the nutritional properties of bread and increasing the fiber content	Alkaline-acid	[171]
Improving the physical qualities of wheat flour dough and bread during refrigeration	-	[172]
Prebiotic activity and antioxidant action	Used as provided	[79]
Cryoprotective effect on lactic cultures of Lactobacillus spp.	Autoclavating at 121 °C	[173]
Yogurt formulation with up to 0.3% β-glucan	Used as provided	[174]
Thickening agent in low-fat yogurt	Used as provided; Alkaline	[144,175,176]
Fortifying role by strengthening the k-carrageenan network	Alkaline-acid	[177]

## Data Availability

Not applicable.
